# Multiple Giant Splenic Artery Aneurysms Causing Sinistral (Left-Sided) Portal Hypertension

**DOI:** 10.1155/2016/6278452

**Published:** 2016-03-27

**Authors:** Kemal Beksac, Derya Karakoc

**Affiliations:** ^1^General Surgery Department, Dr. A. Y. Ankara Oncology Hospital, 06230 Ankara, Turkey; ^2^General Surgery Department, Hacettepe University, 06230 Ankara, Turkey

## Abstract

*Background*. Splenic artery aneurysm is the most common type of visceral aneurysms. They are usually asymptomatic and have a potential for rupture and therefore life-threatening hemorrhage. It is rare for them to cause sinistral portal hypertension.* Case Report*. A 23-year-old female patient presented to our clinic with gastric varices, splenomegaly, pancytopenia, and normal liver functions. She was thus diagnosed with left-sided portal hypertension. Radiologic evaluation showed splenomegaly, splenic vein obstruction, and multiple aneurysms along the splenic artery ranging from 2.5 cm to 7 cm. Splenic artery aneurysm was thought to be the cause of portal hypertension and hypersplenism. We decided splenectomy is the best course of treatment. Pancytopenia could not be corrected preoperatively despite the transfusion treatment. Surgical exploration revealed multiple aneurysms deeply embedded in pancreas. Thrombocyte and erythrocyte transfusion was performed after splenic artery ligation to correct pancytopenia before further intervention. Splenic artery, spleen, and distal pancreas were resected en bloc. Patient's blood parameters became normal within first postoperative day. Patient had an uneventful postoperative course and was discharged without incident.* Conclusion*. Splenic artery aneurysms are rare but potentially life-threatening incidents. Therefore, it is important to know the unusual presentations and prepare accordingly.

## 1. Introduction

Splenic artery aneurysm is the most common type of visceral aneurysms and makes up to 60% of this group [1]. There is a 4 : 1 female-to-male predominance. It has a potential for rupture and erosion, which may cause life-threatening hemorrhage [2]. Stanley et al. report that 22% of cases present as emergencies and 8.5% result in death [3]. They are usually between 2 and 4 cm, saccular, asymptomatic, and present in mid to distal 1/3 of splenic artery [4]. Symptomatic cases, enlarging aneurysms, aneurysms larger than 2 cm, pregnancy, and liver transplantation candidates are considered for treatment [5]. Surgical treatment for splenic artery aneurysms includes splenectomy and aneurysmectomy. Distal pancreatectomy may be performed when the aneurysm is deeply embedded in the pancreatic tissue [6]. Other treatment options include aneurysm ligation, endovascular embolisation, or stenting and revascularisation.

Sinistral (left-sided) portal hypertension is a clinical syndrome in the setting of splenic vein thrombosis due to a primary pancreatic pathology. Splenic artery aneurysm causing sinistral portal hypertension is very rare and is due to splenic vein thrombosis that develops secondary to compression by the aneurysm [7]. Also, if the aneurysm rises from proximal splenic artery, it can cause compression at splenoportal confluence and proximal portal vein directly [8]. The main danger of sinistral portal hypertension is gastric variceal hemorrhage. Splenectomy with treatment of primary pancreatic pathology is the mainstay of management of this disease.

We are reporting a case where this disease represented itself in an unusual way.

## 2. Case Report

**Figure 1:**
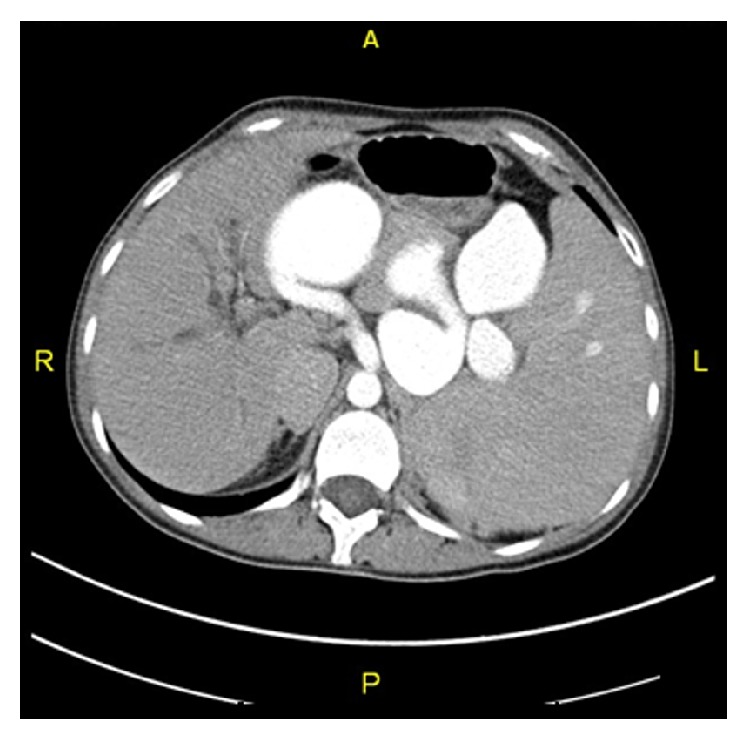
Computerized Tomography (CT) scan shows multiple aneurysms along splenic artery.

**Figure 2:**
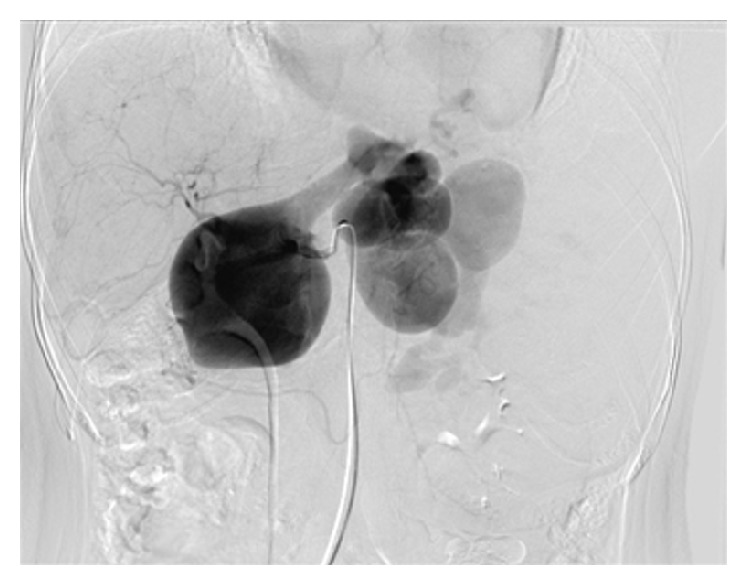
Selective angiography shows the multiple aneurysms along splenic artery.

A 23-year-old female patient presented to emergency room with epilepsy. Her physical examination revealed splenomegaly. She did not have ascites. Initial thrombocyte value was 11000/*μ*L, leukocyte was 2900/*μ*L, and hemoglobin value was 7.8 g/dL. Liver function tests were normal. Cranial Magnetic Resonance Imaging and Echocardiography were normal. Computerized Tomography showed a splenomegaly of 17 cm and multiple aneurysms along the splenic artery ranging from 2.5 cm to 7 cm (Figure 1). Upper gastrointestinal endoscopy revealed isolated gastric varices. Selective angiography revealed multiple aneurysms along splenic artery (Figure 2). Venous phase angiography confirmed the obstruction of splenic vein. Splenic artery embolisation was attempted but it was not successful.

It was learned that she received portal hypertension diagnosis at the age of 9 at a different institution. She was managed at this institution and her file was missing. Patient's parents' description of that period led us to believe that she had been diagnosed with idiopathic portal hypertension since it is occasionally concomitant with huge splenic artery aneurysm and it is difficult to diagnose left-sided portal hypertension at that age. Of course we cannot say for sure what caused the portal hypertension. She dropped out from her treatment 7 years ago and has not received any treatment after that.

Patient was thought to have left-sided portal hypertension due to splenomegaly, normal liver functions, and gastric varices. Since splenic vein was obstructed by the aneurysm, we decided splenectomy and aneurysmectomy were the best course of treatment. Pancytopenia could not be corrected preoperatively despite multiple transfusions. It was evident that pancytopenia could not be corrected without surgical intervention. Surgery was planned.

**Figure 3:**
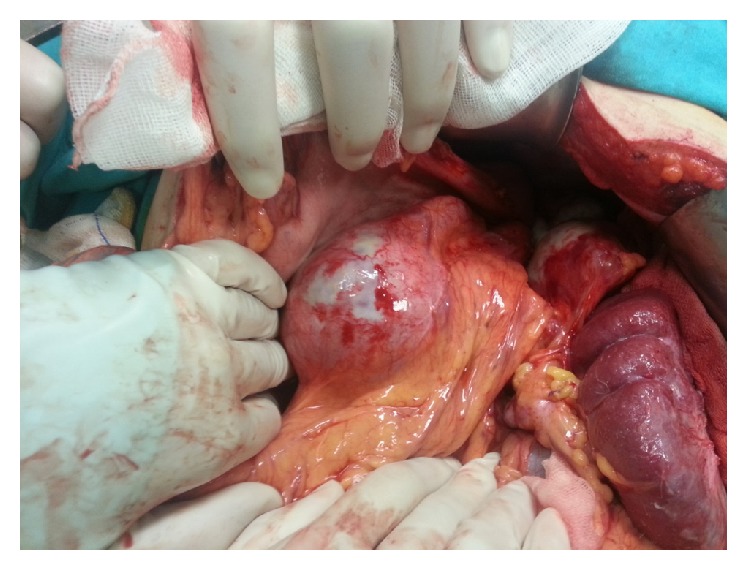
Display of the largest of aneurysms embedded in pancreas.

Surgical exploration revealed multiple aneurysms were embedded in pancreas (Figure 3). First course of action was to ligate the splenic artery. Then thrombocyte and erythrocyte transfusions were performed. Splenic artery, spleen, and distal pancreas were resected en bloc. Patient's thrombocyte values on first postoperative day were 184000/*μ*L, leukocyte value was 4200/*μ*L, and hemoglobin value was 9.7 g/dL. Patient was discharged without incident.

## 3. Discussion

True splenic artery aneurysms are rare entities with an estimated prevalence of 0.02%–0.1%. Giant aneurysms are even rarer with around 20 cases reported so far [9]. Largest reported so far is a pseudoaneurysm of 18 cm [10]. Splenic artery aneurysms are mostly asymptomatic and diagnosed incidentally [11]. Any aneurysm, if left untreated, has a potential for rupture and therefore life-threatening hemorrhage. The life time risk of rupture is 2–10% for small and 28% for giant aneurysms [2]. The highest incidence of rupture is in young pregnant women with mortality being 75% and fetal mortality being 95% [12].

It is generally accepted that all patients with symptomatic aneurysms, patients who are pregnant or in the child bearing age group, patients with lesions larger than 2 cm, patients with lesions gradually increasing in size, or patients with portal hypertension should undergo treatment [11]. Although there are different methods of treatment, surgery is the best option for dealing with giant aneurysms.

Splenic artery aneurysm causing sinistral portal hypertension is rare and is due to splenic vein thrombosis that develops secondary to compression by the aneurysm. There are only few cases reported in literature. Elamurugan et al. [7] report a 22-year-old pregnant patient who had an aneurysm close to splenic hilum and was treated with splenectomy and aneurysmectomy. Debnath et al. [8] report a relatively large 5 × 4 cm juxta-ostial splenic artery aneurysm compressing splenoportal confluence and therefore causing portal hypertension. The case we reported is significantly different in that there are multiple giant aneurysms covering whole splenic artery.

## 4. Conclusion

Splenic artery aneurysms are rare but potentially life-threatening incidents. Therefore, it is important to know of the unusual presentations and prepare accordingly.
